# Role of the IL-33-ST2 axis in sepsis

**DOI:** 10.1186/s40779-017-0115-8

**Published:** 2017-02-02

**Authors:** Hui Xu, Heth R. Turnquist, Rosemary Hoffman, Timothy R. Billiar

**Affiliations:** 1 Department of Surgery, University of Pittsburgh, Pittsburgh, PA 15213 USA; 2 State Key Laboratory of Oral Diseases, Department of Orthodontics, West China Hospital of Stomatology, Sichuan University, Chengdu, 610041 China; 3 Thomas E. Starzl Transplantation Institute, University of Pittsburgh School of Medicine, Pittsburgh, PA 15213 USA

**Keywords:** Sepsis, Interleukin-33, ST2

## Abstract

Sepsis remains a major clinical problem with high morbidity and mortality. As new inflammatory mediators are characterized, it is important to understand their roles in sepsis. Interleukin 33 (IL-33) is a recently described member of the IL-1 family that is widely expressed in cells of barrier tissues. Upon tissue damage, IL-33 is released as an alarmin and activates various types of cells of both the innate and adaptive immune system through binding to the ST2/IL-1 receptor accessory protein complex. IL-33 has apparent pleiotropic functions in many disease models, with its actions strongly shaped by the local microenvironment. Recent studies have established a role for the IL-33-ST2 axis in the initiation and perpetuation of inflammation during endotoxemia, but its roles in sepsis appear to be organism and model dependent. In this review, we focus on the recent advances in understanding the role of the IL-33/ST2 axis in sepsis.

## Background

Sepsis remains a leading cause of mortality in the Intensive Care Unit (ICU) [1]. Accumulating evidence indicates that the IL-33-ST2 axis is involved in the initiation and progression of inflammatory diseases, including sepsis [2–5]. In this review, we provide an update on recent advances on IL-33-mediated immunoregulation in sepsis.

## Definition and epidemiology of sepsis

Sepsis is generally viewed as a condition of overwhelming systemic inflammation in response to an infection that can lead to multiple organ dysfunction [1]. Sepsis is now defined as life-threatening organ dysfunction caused by a dysregulated host response to infection [6], which replaces the term “severe sepsis” [7]. Septic shock occurs when sepsis is complicated by profound circulatory, cellular, and metabolic abnormalities, with a greater risk of mortality than with sepsis alone [6]. The number of cases of severe sepsis is on the rise and now comprises approximately 10–14% of admissions in intensive care units [8–10]. In the United States, the average annual age-adjusted incidence of sepsis is estimated to range between 300 and 1000 cases per 100,000 persons [11].

Sepsis is a leading cause of mortality in the ICU worldwide [1, 12]. Although significant advances in intensive care treatment and organ support have improved outcomes [13, 14], severe sepsis (previous definition) remains associated with mortality rates of 25–30% that increase to 40–50% when septic shock is present [15]. Mortality rates are directly related to the number of organs failing and contributory factors include disseminated intravascular coagulation, derangements of endocrine systems and/or energy metabolism [16]. The prognosis is worse in the elderly, immunocompromised, and critically ill patients [16].

## Pathophysiology of sepsis

Sepsis develops when the host inflammatory response to an infection is exaggerated and subsequently dysregulated [16, 17]. Proinflammatory and anti-inflammatory responses comprise two parallel and overlapping responses during sepsis progression. Excessive inflammation, or sustained immune suppression, is highly correlated to sepsis outcomes [8, 16].

The host response to pathogens is mediated through both innate and adaptive immune systems [7]. The innate immune response functions as the “first line of defense” by immediately responding to invading pathogens in the initiation of sepsis, while the adaptive immune system is comprised of highly specialized cells that respond in a more focused fashion to foreign antigens and are able to develop immunological memory to microbial antigens [7, 16, 18]. Engagement of pattern recognition receptors (PRRs) on both immune and non-immune cells is recognized as the fundamental molecular mechanism of sepsis pathophysiology [8, 16]. Upon pathogen invasion, Toll-like receptors (TLRs) and other PRRs initiate the immune response after the recognition of conserved motifs expressed by pathogens, named pathogen-associated molecular patterns (PAMPs), such as lipopolysaccharide (LPS), lipopeptides, lipoteichoic acid, flagellin, and bacterial DNA [16, 19–21]. TLRs also are triggered by endogenous danger signals, termed danger-associated molecular patterns (DAMPs), which are released from the damaged host tissue after trauma or stress. Identified DAMPs include high mobility group box 1 (HMGB-1), mitochondrial DNA, and S100a proteins [8, 19, 22]. LPS, also known as endotoxin, is among the most potent of all the PAMP molecules [19]. The LPS-dependent TLR4 and caspase-11 (caspase-4/5 in humans) cascades leads to the upregulation of pro-inflammatory/anti-inflammatory mediator production, pyroptotic cell death, and immune dysfunction [16, 23–25].

It has been proposed that the initial hyperactivation of the immune response is followed or overlapped by a prolonged state of immunosuppression, which renders the host susceptible to nosocomial infections [7, 16]. These infections often involve multidrug-resistant bacterial, viral and fungal pathogens [16, 19] and are thought to play a dominant role in the pathogenesis of sepsis-induced multiple organ failure and death [7, 16, 19]. Sepsis-associated immune suppression is thought to result from immune effector cell apoptosis, endotoxin reprogramming, suppressed antigen presentation, increased expression of negative costimulatory molecules and the production of anti-inflammatory cytokines, including type 2 cytokines [16, 19].

A variety of immune cells function differently as sepsis progresses. Macrophages and other cells of the innate immune system release proinflammatory mediators such as IL-1β, IL-6, IL-8, TNF-α, IFN-γ and monocyte chemoattractant protein (MCP)-1 [7, 26–28]. Neutrophils become activated and release the proinflammatory mediators myeloperoxidase (MPO) and proteases [29]. Host cells can also undergo pyroptosis and release large quantities of IL-1α, HMGB-1, and eicosanoids [30–32]. Neutrophil extracellular traps (NETs) released by polymorphonuclear neutrophils (PMNs) are important for anti-microbial defenses but may also propagate inflammatory responses [33]. Th17 cells augment the proinflammatory responses by producing IL-17A, which promotes the production of IL-1β, TNF-α and IL-6 [34]. Macrophages and neutrophils also play immuno-regulatory roles by producing IL-10 and TGF-β [35]. The early upregulation of Th1 responses (characterized by TNF-α, IFN-γ and IL-12 production) gives way to a Th2-dominated response (characterized by IL-4, IL-5, IL-10 and IL-13 production). A shift in the balance from Th1 to Th2 cytokines can cause immune suppression as sepsis progresses [7, 36]. A small subset of CD4^+^ CD25^+^ Foxp3^+^ T cells, referred to as regulatory T cells (Tregs), are upregulated and release IL-10 and TGF-β, favoring Th2 cell proliferation, activation, and differentiation [37]. These cells, along with the upregulation of myeloid-derived suppressor cells and massive immune cell death, are also thought to contribute to the immunosuppressed state [38, 39].

However, our understanding of how inflammatory pathways are modulated to culminate in immune dysfunction during sepsis is far from complete. Likewise, the roles of more recently described immune mediators need to be incorporated into this evolving paradigm. One such mediator is interleukin-33 (IL-33) and its receptor ST2. In this review, we will discuss the current understanding of the role of IL-33 and its regulatory targets in the host response during sepsis.

## Immunobiology of IL-33 and ST2

**Fig. 1:**
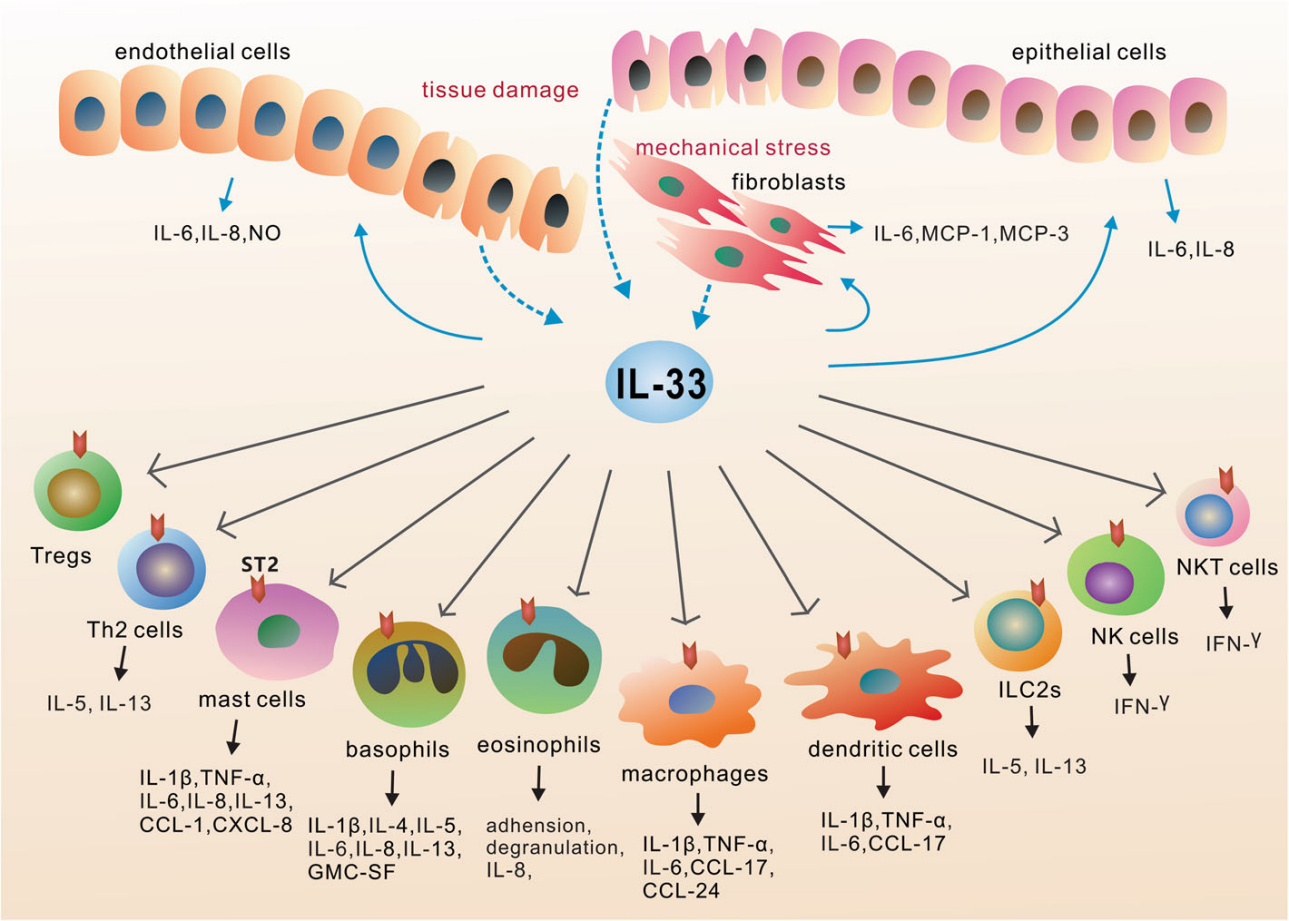
Cellular sources and cellular targets of IL-33. IL-33 is released from endothelial cells, epithelial cells and fibroblasts in response to tissue damage and/or mechanical stress (*indicated as dotted arrow*). After release, IL-33 functions as an alarmin and activates various types of cells (*indicated as solid arrow*), including Th2 cells, Tregs, basophils, mast cells, eosinophils, macrophages, dendritic cells, innate lymphoid cells (ILC2s), NK cells and NKT cells. These cells respond to IL-33/ST2 signaling by producing both pro-inflammatory and anti-inflammatory mediators depending on the immune context in different tissues and diseases

IL-33 was first discovered in 2003 as a nuclear factor from high endothelial venules [40]. In 2005, Schmitz et al. [41] identified IL-33 as a member of the IL-1 family and a ligand for the orphan receptor ST2 (also known as IL-1RL1). IL-33 is mainly produced by structural and lining cells, such as endothelial cells, epithelial cells and fibroblasts, that constitute the first line of host defense against pathogens (Fig. 1) [2, 42–44]. Rodent immune cells, such as macrophages and dendritic cells have been shown to produce IL-33 during allergic inflammation and infection [45–47]. Under homeostatic conditions, endogenous IL-33 is constitutively expressed in the nucleus of cells and can associate with chromatin by binding histones H2A/H2B, though its nuclear roles remain obscure [47, 48]. Full length IL-33 is bioactive, although it can also be processed by proteases (cathepsin G, elastase) into shorter hyperactive forms [47]. Upon tissue damage (necrotic cell death, cell stress) and/or mechanical injury, IL-33 expression increases and it is released into the extracellular space [47]. After release, IL-33 “sounds the alarm” in the immune system by targeting various immune cell types, including T cells, basophils, eosinophils, mast cells, innate lymphoid cells, dendritic cells and macrophages (Fig. 1) [2, 3, 49, 50]. IL-33 was thus proposed to act as an alarmin to sense damage and alert neighboring cells and tissues following infection or trauma and therefore has the potential to influence a broad range of diseases [3–5, 51].

The IL-33 receptor ST2, first identified in 1989, is a member of the IL-1 receptor (IL-1R) family [52]. Through alternative splicing, the ST2 gene encodes two major protein isoforms, a transmembrane full-length form ST2 (ST2 or ST2L) and a soluble, secreted form ST2 (sST2) [3, 50]. sST2 lacks transmembrane and intracellular domains and acts as a decoy receptor for IL-33 [3, 53]. With a nearly undetectable level in normal conditions, the serum concentration of sST2 is increased in patients with pathogenic inflammation, such as asthma [54], autoimmune diseases [55], idiopathic pulmonary fibrosis [56], heart failure [57], and transplant rejection [58]. Membrane-bound ST2 is the functional component for IL-33 signaling [3, 50]. It can be expressed on human and mice CD4^+^ and CD8^+^ T cells, group 2 innate lymphoid cells (ILC2s), mast cells, basophilic and eosinophilic granulocytes, monocytes, dendritic cells, NKT cells and mice NK cells [3, 59]. Recently, it was also reported to be expressed by endothelial cells [60, 61], epithelial cells [62] and fibroblasts [63], thus pointing to the potential importance of IL-33/ST2 signaling in various types of tissues during the pathophysiology of numerous diseases (Fig. 1).

## IL-33/ST2 signaling

**Fig. 2:**
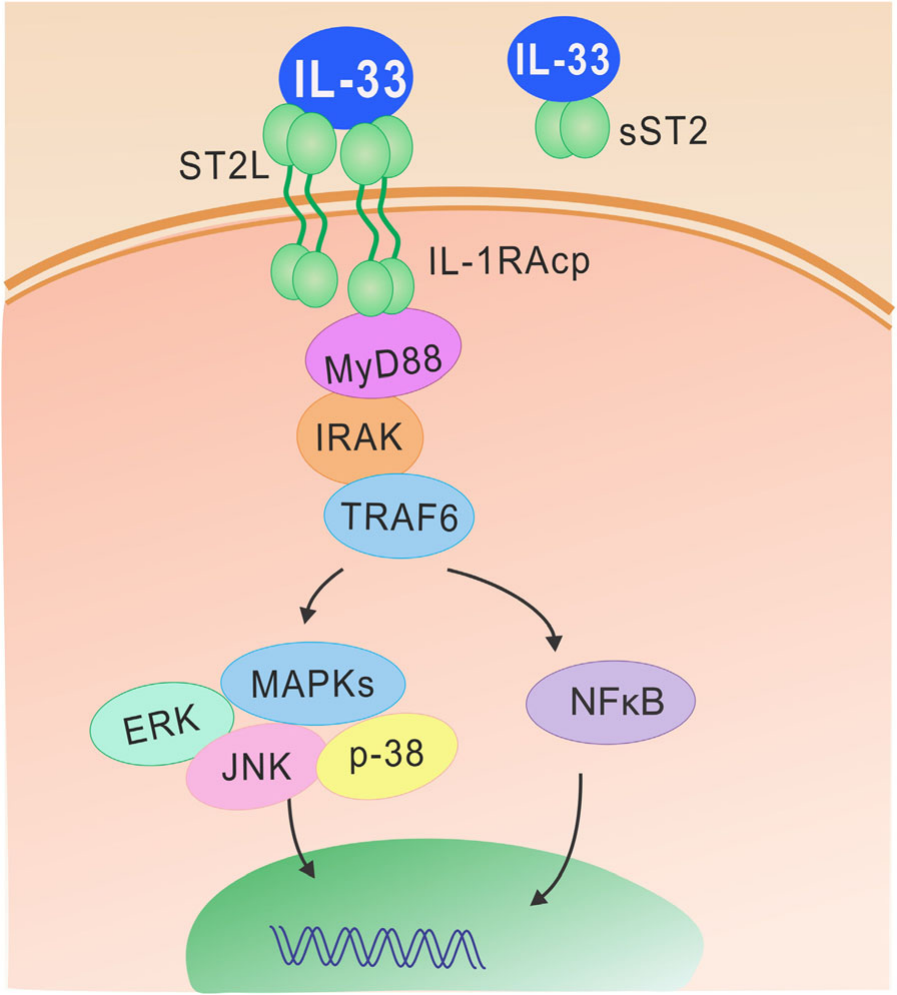
IL-33/ST2 signaling. The binding of IL-33 to ST2 results in the activation of IL-33 bioactivities *via* intracellular pathways, while sST2 acts as a decoy receptor for IL-33

IL-33 binds a heterodimeric receptor complex consisting of ST2 and IL-1R accessory protein (IL-1RAP) and induces the recruitment of myeloid differentiation primary response protein 88 (MyD88), IL-1R-associated kinase (IRAK)-1 and IRAK-4 to the receptor domain in the cytoplasmic region of ST2 (Fig. 2), leading to the activation of downstream signaling, including nuclear factor-kappaB (NF-κB) and MAP kinases (ERK, p38 and JNK) [3, 50]. This subsequently induces the production of various pro- or anti-inflammatory mediators such as IL-6, TNF-α, IL-1β, IL-5 and IL-13 (see below in detail) [3, 50]. IL-33 was proposed to be a multifunctional protein, with reported roles in driving both Th1 and Th2 immune responses depending on type of cells activated, the specific microenvironment and the immune context in different diseases [3, 4].

## Cellular targets of IL-33

### Th1 and Th2 CD4^+^ T cells

The role of IL-33 was first reported in T cells [41]. Naive T cells respond to IL-33 by producing Th2-associated cytokines IL-4, IL-5 and IL-13 in vivo, leading to histopathological changes in the lungs and gastrointestinal tract [41]. IL-33 polarizes murine and human naive CD4^+^ T cells to produce IL-5, and promotes airway inflammation independent of IL-4 [64]. Recently, Villarreal et al. [65, 66] challenged the prevailing opinion that IL-33 strictly targets Th2 CD4^+^ T cells, as they show that IL-33 also has the potential to effect Th1 cell-mediated T cells. Both isoforms of IL-33 (proIL-33 and mtrIL-33) can function as immunoadjuvants to induce profound Th1 CD4^+^ and CD8^+^ T cell responses [65, 66].

### Tregs

Tregs express ST2 and respond to IL-33 by the profound expansion in a ST2-dependent manner [67–69]. IL-33 mediates the Treg-dependent promotion of cardiac allograft survival [69]. IL-33-expanded Tregs protect recipients from acute graft-versus-host disease by controlling macrophage activation and preventing effector T cell accumulation [70]. The protective effects of IL-33-mediated Treg responses were also reported in muscle regeneration [71], hepatitis [72] and colitis [73, 74].

### Mast cells, basophils and eosinophils

IL-33 is a potent inducer of pro-inflammatory mediators by mast cells [75–77]. IL-33 stimulates the production of pro-inflammatory cytokines and chemokines (IL-6, IL-1β, TNF-α, IL-8, IL-13, CCL1 and CXCL8) from human mast cells [78], and synergizes with IgE to promote cytokine production [79, 80]. IL-2 production by IL-33-stimulated mast cells promotes Treg expansion, thus suppressing papain-induced airway eosinophilia [81].

Human basophils express high levels of ST2 receptor and respond to IL-33 with increased production of IL-1β, IL-4, IL-5, IL-6, IL-8, IL-13 and granulocyte macrophage colony-stimulating factor (GMC-SF) [82]. IL-33 synergistically enhances IgE-mediated basophil degranulation [83, 84]. IL-33 potently induces eosinophil degranulation and production of IL-8 and superoxide anion [85], and also enhances eosinophil adhesion and increases eosinophil survival [85, 86].

### Macrophages and dendritic cells

IL-33 enhances the LPS-induced secretion of TNF-α, IL-6, and IL-1β by mouse macrophages [87]. In the setting of allergic airway inflammation, IL-33 amplifies the IL-13-mediated polarization of alternatively activated macrophages and enhances their production of CCL17 and CCL24 [88]. Dendritic cells (DCs) are activated by IL-33 and drive a Th2-type response in allergic lung inflammation [89]. IL-33-activated DCs promote IL-5 and IL-13 production from naive lymphocytes [89, 90]. IL-33 can also activate DCs to produce IL-6, IL-1β, TNF, CCL17 [89] and to express increased levels of CD40, CD80, OX40L, CCR7, MHC-II and CD86 [90]. DCs secrete IL-2 in response to IL-33 stimulation and are required for IL-33-mediated in vitro and in vivo Treg expansion [91].

### Group 2 innate lymphoid cells

Group 2 innate lymphoid cells (ILC2s, previously named natural helper cells, nuocytes, or Ih2 cells) were recently described as members of the ILC family, characterized by the expression of lymphoid markers and type 2 cytokines production, linking the innate and adaptive responses in type 2 immunity in various diseases [92, 93]. ILC2s constitutively express ST2 and respond rapidly to IL-33 with increased proliferation and cytokine production after an allergen challenge or helminth infection [94–97]. IL-33/ST2 signaling is required for IL-5 and IL-13 production from lung ILC2s and airway eosinophilia independent of adaptive immunity [98]. IL-33-dependent IL-5 and IL-13 production from ILC2s can also promote cutaneous wound healing, acting as an important link between the cutaneous epithelium and the immune system [99]. IL-33 protects against experimental cerebral malaria by driving the expansion of ILC2s and their production of IL-4, IL-5 and IL-13 [100] and is required for ILC2-derived IL-13- but not IL-4-driven Type 2 responses during hookworm infection [101]. It also mediates influenza-induced airway hyper-reactivity via an IL-33-ILC2-IL-13 axis [97].

### CD8^+^ T cells, NK and NKT cells

Cytotoxic CD8^+^ T cells can also express ST2 and respond to IL-33. IL-33 synergizes with TCR and IL-12 to augment IFN-γ production from effecter CD8^+^ T cells [102]. IL-33 enhances the production of IFN-γ by both iNKT and NK cells via cooperation with IL-12 [103].

### Endothelial cells, epithelial cells and fibroblasts

IL-33 regulates the activity of many nonimmune cells. Both epithelial cells and endothelial cells produce IL-6 and IL-8 in response to IL-33 [62]. IL-33 promotes nitric oxide production from endothelial cells via the ST2/TNF receptor-associated factor 6 (TRAF6)-Akt-eNOS signaling pathway, leading to enhanced angiogenesis and vascular permeability [61]. Murine fibroblasts respond to IL-33 by producing MCP-1, MCP-3 and IL-6 in a TRAF6-dependent manner [63].

## The role of IL-33/ST2 in sepsis

### Clinical data — serum sST2 levels in sepsis patients

Several studies have shown that IL-33 or sST2 levels are elevated in the circulation of patients with sepsis. Children have significantly higher serum levels of IL-33 and sST2 on the first day of sepsis, raising the possibility that sST2 levels may be useful in the diagnosis of childhood sepsis [104]. On admission [105] and within 24-48 hours of the diagnosis of sepsis [106], adults have significantly higher serum sST2 levels than healthy controls and demonstrate sustained increases in serum sST2 levels during the clinical course of sepsis [106]. Serum sST2 levels correlate with cardiac dysfunction [107], sepsis severity and mortality [106, 107]. In-hospital mortality was higher among patients with elevated serum concentrations of sST2 (above 35 ng/ml) [107]. Parenica et al. [108] concluded that sST2 levels are not a suitable prognostic marker for patients with sepsis shock because ST2 levels failed to predict three-month mortality following sepsis. However, serum concentrations of sST2 are significantly higher in patients with septic shock compared with cardiogenic shock at admission, suggesting the sST2 levels may be useful in identifying patients with sepsis as the etiology of shock in the early phases [108].

### Experimental studies — role of IL-33/ST2 in endotoxemia

The role of the IL-33-ST2 axis has been extensively studied in experimental endotoxemia. Even before the identification of IL-33, it was demonstrated that the ST2 receptor functions as a negative regulator of TLR4 signaling and maintains LPS tolerance [109]. In these studies, ST2-deficient mice did not develop endotoxin tolerance [109]. Specifically, Liu et al. [110] found that ST2 also negatively regulates TLR2 signaling but is not required for bacterial lipoprotein-induced tolerance. A plausible explanation for these differences may lie in the unique signaling transduction and molecular mechanisms of TLR4-mediated tolerance (LPS tolerance) vs TLR2-mediated tolerance (BLP tolerance). Despite the implicated roles of ST2 in endotoxin tolerance, IL-33 triggering of ST2 failed to induce LPS desensitization but instead enhanced the LPS-induced proinflammatory cytokine production (IL-6, TNF-α and IL-1β) in mouse macrophages [111]. This effect is ST2 dependent, as it was not observed in ST2 knockout mice [111]. IL-33 treatment increases macrophage expression of the MD2/TLR-4 components of the LPS receptor as well as levels of the soluble form of CD14, and preferentially affects the MyD88-dependent pathway downstream of TLR-4 and TLR-2, which may explain the enhanced LPS responses of macrophages [111]. These conflicting results indicate distinct roles for IL-33 and ST2 in the pathogenesis of LPS responses. Oboki et al. [112] also found different immune responses between ST2-deficient mice and soluble ST2-Fc fusion protein-treated mice. Taken together, these studies show that the IL-33/ST2 pathway is activated during endotoxemia and plays regulatory roles at the level of endotoxin sensing and signaling. However, more work is required to understand the full range of IL-33 and ST2 actions as regulators or effectors during PAMP exposure.

Apart from the enhanced macrophage responses to LPS as mentioned above, other researchers also reported important roles for IL-33 in macrophage activation for host defenses and proinflammatory responses [113, 114]. IL-33 directly activated bone marrow-derived macrophages (BMDMs) by increasing their expression of MHC class I, MHC class II, CD80/CD86, and inducible NO synthase (iNOS) in a dose-dependent manner and augmented the LPS-induced expression of proinflammatory mediators (e.g., iNOS, IL-6 and TNF-α) in macrophages [113]. Ohno et al. [114] produced results in support of this concept by reporting that exogenous IL-33 potentiated LPS-induced IL-6 production by macrophages and that this effect was suppressed by the blockade of endogenous IL-33 by anti-IL-33 neutralizing antibodies.

In light of the role of IL-33 in LPS-induced proinflammatory responses, researchers have also explored the immunomodulatory functions of sST2, the decoy receptor of IL-33, in LPS-mediated inflammation [115–117]. sST2 treatment inhibited the production of LPS-induced proinflammatory cytokines (IL-6, IL-12 and TNF-α) from BMMs and negatively regulated the expression of TLR-4 and TLR-1 [115]. Consistent results were obtained in vivo after LPS challenge; sST2 administration significantly reduced LPS-mediated mortality and serum levels of IL-6, IL-12, and TNF-α [115]. sST2 down-regulates LPS-induced IL-6 production from a human monocytic leukemia cell line via the suppression of NF-κB binding to the IL-6 promoter [116], and sST2 can be internalized into dendritic cells and suppresses LPS signaling and cytokine production in human monocyte-derived dendritic cells without attenuating the LPS-induced dendritic cell maturation [117]. Conversely, the inhibition of endogenous ST2 through the administration of anti-ST2 antibody aggravated the toxic effects of LPS [115], suggesting distinct roles for IL-33 and ST2 signaling in LPS-induced responses.

The production of IL-33 in the lung was reported in airway inflammation [118] and virus infection [119]. In a mouse model of LPS-induced acute lung injury, the administration of engineered human adipose tissue-derived mesenchymal stem cells (hASCs) overexpressing murine sST2 led to the local suppression of IL-33 signaling and the reduced expression of IL-1β and IFN-γ in the lungs. This was associated with a substantial decrease in lung airspace inflammation, inflammatory cell infiltration and vascular leakage [120]. Yin et al. [121] found that sST2 reduces inflammatory cell infiltration and alveolar hemorrhage in the alveolar airspace and remarkably suppresses proinflammatory cytokine production (TNF-α, IL-6) and TLR-4 gene expression in lung tissues. Taken together, these in vivo studies show that IL-33 signaling can be proinflammatory in the lung during endotoxemia.

### Experimental research — the role of IL-33/ST2 in infection models

Our understanding of the contributions of IL-33 and ST2 during infections is advancing; however, the roles appear to be time, tissue, and model dependent. For example, the effects of ST2 in sepsis were different depending on the model and study design. It was proposed that ST2 contributes to immune suppression during sepsis [122]. In a murine model of cecal ligation and puncture (CLP)-induced sepsis, ST2 deletion leads to improved survival and more efficient bacterial clearance in mice challenged with secondary pneumonia [122]. In contrast, ST2-deficient mice showed increased susceptibility to CLP-induced polymicrobial sepsis with increased mortality, impaired bacterial clearance and increased production of proinflammatory cytokines (TNF-α, IL-6), when compared with their wild-type littermates [123]. This was associated with impaired bacterial uptake, phagocytosis, and killing by ST2-deficient phagocytes, which displayed defects in phagosome maturation, NADPH oxidase 2 (NOX2) activity and superoxide anion production in response to bacterial challenge [123]. When exposed to *Streptococcus pneumoniae* or *Klebsiella pneumoniae*, ST2-deficient blood leukocytes and splenocytes produced lower levels of cytokines and chemokines than wild-type cells [124]. ST2-deficient mice challenged with *Streptococcus pneumoniae* have lower bacterial loads in their spleens compared with their wide-type littermates [124].

Exogenous IL-33 was shown to be protective in murine models of CLP-induced sepsis. IL-33 treatment enhanced the neutrophil influx to the site of infection and thus led to more efficient bacterial clearance and reduced mortality in CLP-induced septic mice [125]. This effect was mediated by preserving CXCR2 expression on neutrophils. The chemokine receptor, CXCR2 has a central role in the recruitment of neutrophils and was down-regulated by TLR4 activation during sepsis. IL-33 reversed the down-regulation of CXCR2 and promoted neutrophil recruitment by repressing G protein-coupled receptor kinase-2 (GRK2) expression [125]. The administration of recombinant IL-33 1 h and 6 h after CLP enhanced bacterial clearance and improved the survival of septic mice [126]. At 24 h after CLP, IL-33 attenuated the severity of organ damage and decreased the serum levels of IL-6, IL-10, TNF-α and IFN-γ, the effect of which was likely to be the consequence of improved bacterial clearance [126]. In an acute *Staphylococcus aureus* peritoneal infection model, IL-33 administration facilitated neutrophil recruitment and bacterial clearance, with higher CXCL2 levels in the peritoneum than untreated mice [127]. Thus, one role for IL-33 appears to be supporting PMN-mediated bacterial clearance in the early phases of bacterial sepsis. There is also some suggestion that IL-33/ST2 may drive the delayed immunosuppression of sepsis. However, more studies are needed to draw this conclusion. We have recently shown that IL-33 can drive ILC2 activation and early IL-5-mediated PMN recruitment in the lung in the CLP model (manuscript submitted). This leads to enhanced early lung injury. Therefore, the cost of enhanced PMN infiltration mediated by IL-33 appears to be secondary, remote lung injury.

## Conclusion

Similar to many immuno-regulatory pathways, the IL-33-ST2 axis plays diverse and context specific roles in sepsis (Table 1). These diverse roles arise, at least in part, through the variety of immune cells that can express ST2 and respond to IL-33. Much remains to be elucidated regarding the precise functions and underlying mechanism of the IL-33-ST2 signaling pathway in sepsis. As our understanding advances, it may be possible to target this pathway to promote antimicrobial defenses or to reduce secondary organ damage.

**Table 1:** Roles of IL-33/ST2 in sepsis models

Disease	Role of IL-33/ST2	Referenced
Sepsis	Sepsis patients have higher levels of serum IL-33 and sST2	[104–108]
Endotoxemia	ST2 negatively regulates TLR4 signaling and maintains LPS tolerance	[109]
Endotoxemia	ST2 negatively regulates TLR2 signaling, but is not required for BLP tolerance	[110]
Endotoxemia	IL-33 enhances LPS-induced proinflammatory mediators in mouse macrophages in a ST2-dependent manner	[111, 113, 114]
Endotoxemia	sST2 reduces LPS-mediated mortality and inhibits LPS-induced proinflammatory cytokines	[115–117]
Endotoxemia	sST2 reduces inflammatory cell infiltration and vascular leakage, and suppresses proinflammatory cytokine production in lung tissues	[120, 121]
Abdominal sepsis	ST2 deletion protects mice challenged with secondary pneumonia	[122]
Abdominal sepsis	ST2 deficiency increases the susceptibility to sepsis	[123]
*Streptococcus pneumoniae* infection	ST2 deficiency protects mice challenged with S. pneumonia	[124]
Abdominal sepsis	IL-33 enhances neutrophil recruitment and protects mice with more efficient bacterial clearance and improved survival	[125, 126]
Abdominal sepsis	IL-33 administration attenuates organ damage in the late phase of sepsis	[126]
*Staphylococcus aureus* infection	IL-33 administration facilitates neutrophil recruitment and bacterial clearance	[127]

## Abbreviations

BMDMs: Bone marrow-derived macrophages
CLP: Cecal ligation and puncture
DAMPs: Danger-associated molecular patterns
DCs: Dendritic cells
GMC-SF: Granulocyte macrophage colony-stimulating factor
GRK2: G protein-coupled receptor kinase-2
hASCs: Human adipose tissue-derived mesenchymal stem cells
HMGB-1: High mobility group box 1
ICU: Intensive care unit
IL-1R: IL-1 receptor
IL-1RAP: IL-1R accessory protein
IL-33: Interleukin-33
ILC2s: Group 2 innate lymphoid cells
iNOS: Inducible NO synthase
IRAK: IL-1R-associated kinase
LPS: Lipopolysaccharide
MCP: Monocyte chemoattractant protein
MPO: Myeloperoxidase
MyD88: Myeloid differentiation primary response protein 88
NETs: Neutrophil extracellular traps
NF-κB: Nuclear factor-kappaB
NOX2: NADPH oxidase 2
PAMPs: Pathogen-associated molecular patterns
PMNs: Polymorphonuclear neutrophils
PRRs: Pattern recognition receptors
TLRs: Toll-like receptors
TRAF6: TNF receptor-associated factor 6
Tregs: Regulatory T cells
